# Disparities in SARS-CoV-2 Infection by Race, Ethnicity, Language, and Social Vulnerability: Evidence from a Citywide Seroprevalence Study in Massachusetts, USA

**DOI:** 10.1007/s40615-022-01502-4

**Published:** 2023-01-18

**Authors:** Wilfredo R. Matias, Isabel R. Fulcher, Sara M. Sauer, Cody P. Nolan, Yodeline Guillaume, Jack Zhu, Francisco J. Molano, Elizabeth Uceta, Shannon Collins, Damien M. Slater, Vanessa M. Sánchez, Serina Moheed, Jason B. Harris, Richelle C. Charles, Ryan M. Paxton, Sean F. Gonsalves, Molly F. Franke, Louise C. Ivers

**Affiliations:** 1 Division of Infectious Diseases, Massachusetts General Hospital, 55 Fruit St, BUL-130, Boston, MA 02114 USA; 2 Division of Infectious Diseases, Brigham and Women’s Hospital, Boston, MA USA; 3 Center for Global Health, Massachusetts General Hospital, Boston, MA USA; 4 Department of Global Health and Social Medicine, Harvard Medical School, Boston, MA USA; 5 Harvard Data Science Initiative, Cambridge, MA USA; 6 Department of Medicine, Brigham and Women’s Hospital, Boston, MA USA; 7 Department of Pediatrics, Harvard Medical School, Boston, MA USA; 8 Department of Medicine, Harvard Medical School, Boston, MA USA; 9 Holyoke Board of Health, Holyoke, MA USA; 10 Harvard Global Health Institute, Cambridge, MA USA

**Keywords:** SARS-CoV-2, COVID-19, Disparities, Seroprevalence, Serosurvey, Antibodies

## Abstract

**Objectives:**

Uncovering and addressing disparities in infectious disease outbreaks require a rapid, methodical understanding of local epidemiology. We conducted a seroprevalence study of SARS-CoV-2 infection in Holyoke, Massachusetts, a majority Hispanic city with high levels of socio-economic disadvantage to estimate seroprevalence and identify disparities in SARS-CoV-2 infection.

**Methods:**

We invited 2000 randomly sampled households between 11/5/2020 and 12/31/2020 to complete questionnaires and provide dried blood spots for SARS-CoV-2 antibody testing. We calculated seroprevalence based on the presence of IgG antibodies using a weighted Bayesian procedure that incorporated uncertainty in antibody test sensitivity and specificity and accounted for household clustering.

**Results:**

Two hundred eighty households including 472 individuals were enrolled. Three hundred twenty-eight individuals underwent antibody testing. Citywide seroprevalence of SARS-CoV-2 IgG was 13.1% (95% CI 6.9–22.3) compared to 9.8% of the population infected based on publicly reported cases. Seroprevalence was 16.1% (95% CI 6.2–31.8) among Hispanic individuals compared to 9.4% (95% CI 4.6–16.4) among non-Hispanic white individuals. Seroprevalence was higher among Spanish-speaking households (21.9%; 95% CI 8.3–43.9) compared to English-speaking households (10.2%; 95% CI 5.2–18.0) and among individuals in high social vulnerability index (SVI) areas based on the CDC SVI (14.4%; 95% CI 7.1–25.5) compared to low SVI areas (8.2%; 95% CI 3.1–16.9).

**Conclusions:**

The SARS-CoV-2 IgG seroprevalence in a city with high levels of social vulnerability was 13.1% during the pre-vaccination period of the COVID-19 pandemic. Hispanic individuals and individuals in communities characterized by high SVI were at the highest risk of infection. Public health interventions should be designed to ensure that individuals in high social vulnerability communities have access to the tools to combat COVID-19.

**Supplementary Information:**

The online version contains supplementary material available at 10.1007/s40615-022-01502-4.

## Background

A growing body of literature has documented the disproportionate impact of the coronavirus disease 2019 (COVID-19) pandemic on underserved communities in the USA [1, 2]. In the first year of the pandemic, however, due to limitations in access to testing and the high frequency of asymptomatic infections, official reports underestimated actual infection rates with severe acute respiratory syndrome coronavirus 2 (SARS-CoV-2), the virus that causes COVID-19. As a result, public health officials were left with an incomplete picture of viral spread, associated risk factors, and potential disparities [3, 4]. To address these limitations, seroepidemiologic prevalence studies (serosurveys), which measure the presence of anti-SARS-CoV-2 antibodies as a marker of prior infection, are an important public health tool to estimate incidence and guide public health responses [5, 6].

In the USA, the Commonwealth of Massachusetts (MA), fueled by a super-spreader event of SARS-CoV-2, became one of the early epicenters of the pandemic [7]. Early indications were that Black, Hispanic, and other vulnerable communities were being disproportionately hospitalized, but robust data on the extent to which this was happening and whether it was related to higher infection rates, disparities in testing, social vulnerability, or other unknown factors was missing [8–11]. Holyoke is a post-industrial, majority Hispanic/Latino/Latina city in Massachusetts (53.9%) with high levels of socio-economic disadvantage [12]. Based on 2018 census data, the poverty rate and median household income were 29.7% and $40,656, respectively, compared to 10.8% and $79,835 for MA [13]. The city was disproportionately affected by the first surge of COVID-19 as evidenced by a high initial case count relative to the rest of MA [14, 15]. To fill the gap in our knowledge regarding risk factors for and disparities in SARS-CoV-2 infection and inform local public health responses, we conducted a serosurvey of SARS-CoV-2 antibodies in Holyoke, MA, between November 2020 and January 2021, shortly prior to the second wave of the pandemic in Massachusetts, which at the time was the largest and second deadliest wave in terms of incident cases and deaths, respectively.

## Methods

### Study Design and Sampling

We conducted a representative, cross-sectional household-based SARS-CoV-2 serosurvey in Holyoke, MA. We obtained an official list of the 17,828 addresses in the city’s 11 census tracts from city records. We identified and removed listings corresponding to congregate living facilities, vacant buildings, duplicate entries, and empty listings. The remaining addresses were considered eligible for sampling. We then randomly sampled 2000 addresses from this list expecting a 25% response rate (Supplementary material) [16].

### Participant Recruitment

All study protocols were implemented using Spanish and English materials by bilingual study staff. Participants were enrolled from November 5, 2020, to December 31, 2020. The study team mailed an invitation letter to sampled addresses that contained a QR code and unique ID for an online bilingual survey hosted on REDCap (Research Electronic Data Capture) at Massachusetts General Hospital [17]. Among selected addresses, all individual household members aged ≥ 6 months and residing at the address were eligible. A household was defined as a group of persons who slept under the same roof most nights. Households were given a period of approximately 5 days to respond by either taking the online survey or contacting the study team. Households could opt out of future communication by calling a study phone number. Following that period, data collectors made reminder follow-up calls, where participants had the option of completing the survey over the phone. They also conducted home visits to follow up with households that lacked a telephone number, were unresponsive to calls, or requested a visit from the team. To raise awareness about the study and increase community engagement, the study team distributed fliers and made announcements via Facebook and local media in both English and Spanish.

Following the first month of recruitment, we mailed a second wave of invitation letters to the originally sampled households, excluding households that had either already completed their surveys or opted out of the study. Data collectors made follow-up phone calls to households with incomplete surveys and those who had not mailed back their samples.

### Data Collection

The survey tool consisted of an eligibility and informed consent form, one household-level survey, and individual adult and child surveys for each consenting adult and child household member. Surveys included questions regarding sociodemographic characteristics, occupation and employment history, clinical history, symptoms, COVID-19 testing, and exposure history. Upon completion of these surveys, blood test kits were either mailed to participant addresses or physically provided during home visits. We provided a $25 gift card to each household in which at least one person completed the entire study.

### Sample Collection, Transportation, and Laboratory Testing

Test kits contained supplies and instructions (in English and Spanish) for the self-collection of dried blood spot (DBS) samples. Individuals were instructed to perform a pinprick on the finger using a lancet and apply it to Whatman® Protein Saver 903 filter paper collection cards (https://www.cytivalifesciences.com). Once obtained, samples were return-mailed using a pre-addressed envelope to Massachusetts General Hospital (Boston, MA, USA) where they were stored at − 20°C with desiccant until tested. DBS sampling has been validated for use in antibody testing of SARS-CoV-2 and other pathogens [18–20].

DBS were eluted and tested for the presence of SARS-CoV-2 IgG and IgM receptor-binding domain of the spike protein of SARS-CoV-2 using a quantitative ELISA previously developed and validated using reverse transcriptase polymerase chain reaction (RT-PCR)-positive mild and severe SARS-CoV-2 infections and pre-pandemic samples at Massachusetts General Hospital [20–22]. The test specificity and sensitivity estimates were respectively 99.5% (95% CI 99.0–99.8) and 70.6% (95% CI 61.2–79.3%) for IgG antibodies and were calculated using samples from the Boston area (Supplementary Fig. 1). Further details are provided in Sect. 2 of the  Supplementary Materials (Supplementary Tables 2 and 3).

### Statistical Analysis

Our main SARS-CoV-2 seroprevalence estimate was the proportion of the population that had IgG antibodies detected as this aligns with prior studies [23]. We also calculated seroprevalence estimates with corresponding 95% credible intervals (CI) for the following combinations of IgG and IgM antibody positivity: any IgG, any IgM, IgG only, IgM only, and IgG or IgM.

We used a modified version of the Bayesian procedure proposed by Stringhini et al. [24] to calculate prevalence estimates with corresponding 95% CI’s. The procedure accounted for uncertainty in the antibody test sensitivity and specificity. Within the procedure, a random intercept was used to account for clustering by household, and weighting was applied to ensure the seroprevalence estimates were representative of the population of Holyoke. Details on the procedure are provided in Sect. 1 of the Supplementary Materials.

A raking procedure was used to construct weights based on the distribution of age, race and ethnicity, gender, and census tract in Holyoke from the 2019 American Community Survey (ACS) (Table 1). Notably, gender is reported as the percent of “Female persons” in Holyoke in the ACS survey results, such that we only have two categories for gender: Female and non-Female, which includes male, transgender, and non-binary persons. For the purposes of constructing weights, we collapsed sparse categories of race and ethnicity into a “Grouped category.” The census tracts were also collapsed into “high vulnerability” and “low vulnerability” groups based on the Centers for Disease Control and Prevention (CDC) social vulnerability index (SVI) [25]. The CDC SVI uses 16 US census indicators characterizing four domains (socioeconomic status, household characteristics, racial and ethnic minority status, and housing type/transportation) to generate domain-specific and overall social vulnerability rankings of census tracts relative to each other. These rankings can then be used by local officials to identify communities that may need support during emergencies such as pandemics. For this study, “high vulnerability” was defined as having an SVI greater than the 75th percentile of census tracts in Massachusetts—9 of the 11 census tracts were considered highly vulnerable.

**Table 1:** Unweighted demographic characteristics of survey participants compared with 2019 American Community Survey estimates for the city

Characteristic	Responded to survey		Provided antibody sample		Holyoke City^1^	
	*N* = 472	%	*N* = 328	%	*N* = 40,241	%
Age group (years) median (IQR)^*^						
0–19 14 (816)	50	10.6	27	8.2	10,406	25.8
20–44 33 (27–37.3)	130	27.5	76	23.2	14,335	35.7
45–59 53 (50.3–56)	135	28.6	94	28.7	7607	18.9
60–84 68 (64–73)	149	31.6	123	37.5	7019	17.4
85 and over 88 (86.5–89.3)	8	1.7	8	2.4	874	2.2
Gender						
Female	263	55.7	180	54.9	20,747	51.6
Male	200	42.4	139	42.4	19,494	48.4
Transgender woman	1	0.2	1	0.3	-^+^	-
Transgender man	0	0.0	0	0	-	-
Non-binary	7	1.5	7	2.1	-	-
Prefer not to answer	1	0.2	1	0.3	-	-
Other	0	0.0	0	0	-	-
Race and ethnicity						
Hispanic or Latino/Latina	126	26.7	66	20.1	21,704	53.9
Non-Hispanic or Non-Latino/Latina	346	73.3	262	79.9	18,537	46.1
White	311	65.9	239	72.9	16,636	41.3
Black or African American	10	2.1	5	1.5	1162	2.9
Asian	7	1.5	5	1.5	239	0.6
AIAN^2^	1	0.2	1	0.3	78	0.2
NHPI^3^	0	0.0	0	0.0	0	0.0
Other race	6	1.3	5	1.5	3	0.0
Two or more races	6	1.3	2	0.6	419	1.0
Prefer not to answer	5	1.1	5	1.5	-	-

^1^US Census Bureau (2019). American Community Survey, Demographic, and Housing Estimates. Table DP05

^2^AIAN American Indian or Alaskan Native

^3^NHPI Native Hawaiian and Other Pacific Islander

^*^Median (IQR) for the population of 328 individuals providing an antibody sample

^+^Indicates that this data was not available in the American Community Survey

We compared seroprevalence estimates and 95% CI’s across subgroups. To investigate disparities in known risk factors for SARS-CoV-2 infection, we reported sociodemographic and clinical factors stratified by race and ethnicity. Analyses were conducted in R V4.0.0 using *survey* and *rstan* packages [26, 27].

### Patient Consent Statement

Written informed consent was obtained from adults 18 years or older. Informed parental consent and assent was obtained for children ages 14–17. Parental consent was obtained for children under the age of 14, with documented verbal assent by the caregiver sought for minors between the ages of 7 and 13.

### Ethics Approval Statement

This protocol was reviewed by the Mass General Brigham Human Research Committee Institutional Review Board (Protocol ID: 2020P002560, November 2nd, 2020).

## Results

### Study Population

**Fig. 1:**
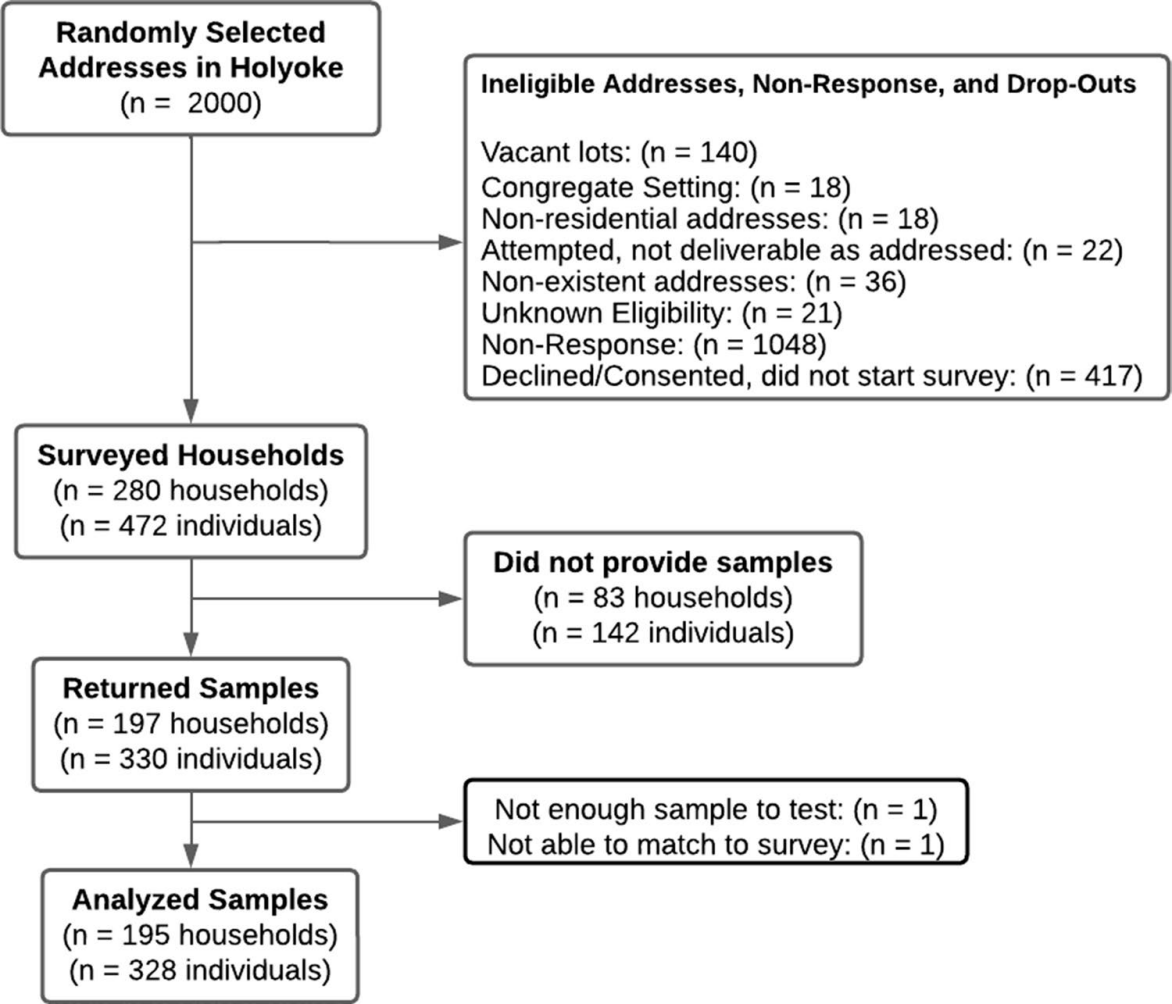
Study participant flow diagram

From the final list of 17,204 addresses, we randomly sampled 2000 and mailed invitation letters and followed up recruitment as described above. Two hundred eighty households (14%) with 472 individuals agreed to participate and completed household and individual-level questionnaires. Figure 1 demonstrates a complete flow diagram of participant progress through study phases. Supplementary Fig. 3 demonstrates timing of survey completion and dried blood spot sample collection. Mean household size was 2.28 individuals (Standard deviation [SD]: 1.2). Within participating households, an average mean of 1.77 household members (SD: 1.03) consented to participate. Of these, 197 households (70.4%) and 330 individuals (69.9%) completed and returned a DBS sample for analysis. A total of 328 samples from 195 households were analyzed. The mean household size for households with individuals that provided a DBS sample and were analyzed was 2.09 individuals (SD: 1.13), and the mean number of individuals per household that consented to participate was 1.71 (SD: 0.96).

Among the 280 households that completed the survey, 37 reported hospitalization of a household member since February 2020 (13.3%, 1 missing response). Only one hospitalization was reported to be a result of COVID-19. There were 2 reported deaths since February 2020 among 277 households (3 missing responses). Two hundred forty-eight of 471 (52.7%, 1 missing) respondents reported being tested for COVID-19 at some point prior to the survey. Twenty-five of 471 respondents (5.31%, 1 missing response) described being diagnosed by a healthcare provider with pneumonia or other respiratory infection (may include COVID-19 diagnosis) since February 2020. Twenty of the 468 respondents (4.3%, 4 missing response) reported testing positive for COVID-19 at least once.

Demographic characteristics including gender, age group, and race and ethnicity breakdown for the entire study population are listed in Table 1. Individuals from younger age groups (0–19 and 20–44 years of age) and individuals identifying as Hispanic/Latino/Latina were underrepresented in the study population relative to the population of Holyoke. We addressed this with a second round of invitation letters and by weighting the Bayesian model.

### Citywide Seroprevalence of SARS-CoV-2 Antibodies

Of 328 individual samples tested, 27 individuals from 20 households were positive for SARS-CoV-2 IgG or IgM antibodies; after adjusting for clustering, differential response rates, and imperfect test sensitivity, this corresponded to a citywide seroprevalence estimate of 13.6% (95%CI 6.7–23.7). Twenty-five individuals were positive for SARS-CoV-2 IgG antibodies, corresponding to an adjusted citywide seroprevalence estimate of 13.1% (6.9–22.3%). Seroprevalence estimates for individuals with IgG only, IgM only, any IgG, any IgM, and IgG or IgM are listed in Table 2. Supplementary Fig. 2 provides seroprevalence estimates under various sensitivity estimates ranging from 60.2 to 78.8%, corresponding to overall IgG seroprevalence estimates ranging from 15.3 to 11.7%, respectively.

**Table 2:** Seroprevalence by antibody positivity profile

Characteristic	No. tested	No. positive	Seroprevalence % (95% CI)
Any IgG	328	25	13.1 (6.9–22.3)
IgG only	328	18	7.8 (3.4–16.4)
Any IgM	328	9	11.0 (2.3–25.9)
IgM only	328	2	0 (0–5.0)
IgG or IgM	328	27	13.6 (6.7–23.7)

### Seroprevalence of SARS-CoV-2 Antibodies by Sociodemographic Characteristics and Exposure History

Seroprevalence estimates were calculated for the following subgroups: age groups, gender, race and ethnicity, social vulnerability index, primary language spoken in the household, and other sociodemographic characteristics (Table 3). Seropositivity varied across multiple subgroups, though credible intervals tended to be wide. The seroprevalence estimate was highest among individuals 20–44 years old (17.6%; 95% CI 7.5–32.4) and decreased with age for ages > 44. The seroprevalence estimate was higher at 16.1% (95% CI 6.2–31.8) among individuals identifying as Hispanic/Latino/Latina compared to a seroprevalence estimate of 9.4% (95% CI 4.6–16.4) among individuals identifying as non-Hispanic white, corresponding to a risk difference of 6.6% (95% CI − 4.3 to 21.8). The seroprevalence estimate among Spanish-speaking households was 21.9% (95% CI 8.3–43.9) compared to 10.2% (95% CI 5.2–18.0) among English-speaking households, with a risk difference of 11.6% (95% CI − 2.3 to 32.2). Individuals living in high vulnerability areas (14.4%; 95% CI 7.1–25.5) had a higher seroprevalence estimate than individuals living in low vulnerability areas (8.2%; 95% CI 3.1–16.9), corresponding to a risk difference of 6.0% (95% CI − 3.6 to 17.5). The seroprevalence among individuals reporting an exposure to a household member was 72.4% (95% CI 32.6–99.7), while only 21.3% (95% CI 8.0–41.5%) among those reporting an exposure to a non-household member and 5.8% (95% CI 1.9–13.4%) among those reporting no known exposure to someone with COVID-19.

**Table 3:** Seroprevalence^1^ by sociodemographic characteristics

Characteristic (*N* if not 328)	*N*^2^	*n*^3^	Weighted^4^ seroprevalence, % (95% CI)
Age groups (years)			
0–19	27	2	11.2 (1.9–32.9)
20–44	76	10	17.6 (7.5–32.4)
45–59	94	6	9.8 (3.4–21.1)
60 and above	131	7	8.9 (3.6–17.4)
Gender			
Female	180	15	14.4 (6.7–26.0)
Grouped categories (male or non-binary)	148	10	11.6 (5.0–22.4)
Race and ethnicity			
Non-Hispanic, White	239	16	9.4 (4.6–16.4)
Hispanic or Latino/Latina	66	8	16.1 (6.2–31.8)
Non-Hispanic, Grouped categories	23	1	7.3 (0.7–24.4)
Social vulnerability index			
High	185	16	14.4 (7.1–25.5)
Low	143	9	8.2 (3.1–16.9)
Primary language spoken in the household			
English	275	18	10.2 (5.2–18.0)
Spanish	36	7	21.9 (8.3–43.9)
Multi-lingual/other	16	0	3.9 (0.1–27.0)
Missing	1	0	
Highest education level (*N* = 305)^*^			
Some high school or less	22	2	12.5 (2.2–34.8)
High school/GED or some college	95	8	15.2 (5.9–29.0)
Associate or bachelor’s degree	101	7	12.6 (4.8–24.7)
Master’s doctorate or professional degree	80	6	11.7 (4.0–23.6)
Missing	7	0	
Employment status on February 1st, 2020 (*N* = 305)^*^			
Working	181	19	18.1 (9.7–29.6)
Not working	94	2	3.9 (0.5–12.7)
Other	29	2	11.5 (1.1–32.0)
Missing	1	0	
Worked outside home during “stay at home” order (*N* = 305)^*^			
No	211	13	10.8 (4.8–20.1)
Yes	93	10	19.5 (9.4–33.8)
Missing	1	0	
Worked as a health worker in a healthcare setting (*N* = 93)^*+^			
No	75	7	13.1 (4.4–27.7)
Yes	18	3	20.7 (4.8–53.4)
Workplace offered paid sick leave, work-from-home, overtime, or hazard pay during Mar–June (*N* = 93)^*+^			
None of the above	31	4	15.3 (3.2–38.7)
1–2 of the above	56	5	13.6 (4.5–30.9)
3–4 of the above	6	1	8.4 (0.3–45.1)
Travel since February 1st, 2020 (*N* = 328)			
No	114	8	12.5 (4.5–25.2)
Yes, to a different city	117	5	6.9 (1.4–16.2)
Yes, to a different state	88	8	10.8 (3.4–26.5)
Yes, to a different country	7	4	55.5 (14.2–98.0)
Missing	2	0	
Annual household income (*N* = 328)			
Less than $15,000	21	6	33.8 (10.6–66.9)
15,000–$50,000	69	1	2.0 (0–14.7)
50,000–$80,000	64	3	7.4 (0.3–22.4)
80,000–$160,000	94	9	15.8 (6.3–29.2)
$160,000	24	4	13.9 (2.7–38.3)
Prefer not to answer	53	2	8.2 (0.1–26.0)
*Missing*	*3*	*0*	
Home type (*N* = 328)			
Single family	189	11	8.4 (3.6–16.1)
Multi-family	67	5	14.0 (4.2–29.4)
Apartment or condominium	67	8	15.9 (5.8–33.4)
Other	3	1	23.1 (2.2–69.8)
Missing	2	0	
Rent or own the home (*N* = 328)			
Someone in the household owns the home	235	16	12.1 (5.6–21.5)
We have a landlord	88	8	13.8 (5.0–29.4)
Missing	5	1	
Number of people living in the household			
One	65	2	4.7 (0.3–14.7)
Two	130	11	13.9 (6.1–26.0)
Three	73	3	3.0 (0.0–17.1)
Four or more	60	9	25.8 (9.2–50.6)

^1^Seroprevalence for demographics groups based on IgG antibody positivity (i.e., at least IgG positive)

^2^* N* refers to the total numbers of individuals in each category

^3^*n* refers to the total number of individuals seropositive for IgG in each group

^4^Weights were computed as the inverse probability of selection and adjusted so that the marginal distribution of age group, gender, race and ethnicity, and social vulnerability index of the sample agreed with population estimates

^*^Among adult respondents only

^+^Among individuals that responded “Yes” to working outside the home during the “stay at home” order

### Sociodemographic, Symptom Testing, and Exposure History by Race and Ethnicity

Compared to non-Hispanic white individuals, individuals identifying as Hispanic/Latino/Latina were younger and had attained lower education levels (Supplementary Table 1). They had higher rates of unemployment at the start of the pandemic and were more likely to have their salary impacted by COVID-19. They were more likely to work at a place that offered no benefits such as paid sick leave or work-from-home and were more likely to use the bus as a means of transportation. Housing conditions were also different, with Hispanic/Latino/Latina individuals more likely to live in apartments or condominiums, rent rather than own their homes, and report a higher density of individuals living in the household (Supplementary Table 1).

## Discussion

We estimated the citywide prevalence of SARS-CoV-2 IgG antibodies to be 13.1% in Holyoke at the end of a second surge of the pandemic in the Commonwealth of Massachusetts between November 2020 and January 2021, prior to widespread vaccination in this community, and shortly before the second wave of the pandemic, which at the time was the largest and second deadliest wave in terms of incident cases and deaths. Several groups demonstrated higher risk of prior infection than their counterparts in the city, based on the presence of SARS-CoV-2 antibodies.

Individuals identifying as Hispanic/Latino/Latina had a higher seroprevalence estimate compared to those identifying as non-Hispanic white, suggesting that these members of the community were at high risk of SARS-CoV-2 infection. Although credible intervals around effect estimates were wide, this finding is consistent with prior studies documenting racial and ethnic disparities in SARS-CoV-2 metrics affecting minoritized communities nationally and in Massachusetts, including disparities in ability to follow non-pharmacologic interventions, testing, infections, hospitalizations, and deaths [2, 9, 28, 29]. Additionally, a nationwide SARS-CoV-2 serosurvey of blood donations around this time period demonstrated that individuals identifying as Hispanic had the highest seroprevalence of SARS-CoV-2 antibodies of all racial and ethnic groups [30]. There are multiple potential mediators of these disparities, lending support to a true difference in risk. In Holyoke, most Hispanic communities live in census tracts characterized by high socioeconomic deprivation [25]. In our study population, compared to non-Hispanic white individuals, individuals identifying as Hispanic reported lower education levels, higher unemployment rates, had lower access to benefits such as paid sick leave or work-from-home, and were more likely to live in high-density housing. Public health responses to COVID-19 and future pandemics should be designed to directly mitigate these risk factors. For example, the provision of financial and social supports would aid individuals in adhering to public health efforts that mitigate disease spread.

Individuals from predominantly Spanish-speaking households, almost all of whom identified as Hispanic/Latino/Latina, had a seroprevalence estimate higher than individuals from English-speaking households. Our experience suggests that the availability of Spanish-language-concordant public health outreach was limited in Massachusetts during the early phases of the pandemic. The absence of linguistically concordant public health interventions may directly impact an individual’s ability to understand and apply preventive guidance and thus mediate SARS-CoV-2 infection risk [31, 32]. Further studies are needed to identify the key mediators of the relationship between race and ethnicity, social vulnerability, language, and risk of SARS-CoV-2 infection, which can then inform public health interventions tailored to these populations.

The overall seroprevalence measured in this study, when interpreted in the context of other seroprevalence studies and routine case-surveillance data, provides insight into the dynamics of the pandemic in the region. In April 2020, a serosurvey using convenience sampling of asymptomatic individuals in the predominantly Hispanic community of Chelsea, MA, demonstrated a seroprevalence of 31.5% [33]. This serosurvey was limited by non-representative convenience sampling that likely resulted in a biased estimate, and the use of a rapid lateral flow immunoassay. Later, between July and August 2020, a university-related population and their household members in Massachusetts demonstrated a lower seroprevalence of 4–5.3% [34]. Our findings in this study conducted several months later are consistent with the increasing number of reported cases during the second surge of COVID-19 in MA. However, the seroprevalence measured in this study was not as high as might be expected approximately 10 months into the pandemic, especially since by various metrics and media reports, Holyoke was one of the most COVID-19-impacted communities in the commonwealth early on in the pandemic [14, 15]. This may be in part due to the impact of decaying antibody titers, the kinetics of which vary depending on the population [35].

We also identified an important testing gap. Overall, the prevalence of any anti-SARS-CoV-2 antibodies (measured by IgG or IgM) in this survey corresponds to a cumulative case count of 5593 compared to the city’s actual case count of 3963 on January 28th, 2021 based on testing by RT-PCR [36]. By this estimate, nearly one third of all SARS-CoV-2 infections in Holyoke were undetected by existing surveillance and screening mechanisms. This level of underascertainment is lower than that demonstrated in other serosurveys throughout the USA, a finding that may be explained by the high availability of testing throughout Holyoke, where two public testing sites were established [37, 38]. However, this discrepancy highlights an important testing gap that should be addressed as we continue to respond to ongoing outbreaks of SARS-CoV-2. Missed infections, especially in a community that is already socially vulnerable, can result in delays in testing and appropriate care, and individuals being overlooked when public health resources are distributed.

In our study, seroprevalence was higher among individuals reporting a COVID-19 exposure that was a household member compared to a COVID-19 exposure to a non-household member. This finding corroborates the importance of intra-household exposures in the control of COVID-19 [39]. Given the role of intra-household transmission, public health interventions and resources should be targeted to preventing transmission in the household, such as providing isolation and quarantine sites outside the home, PPE for individuals taking care of sick family members, timely and sequential testing for exposed household members, and guidance on how to safely distance within the home.

This study has several limitations including the small sample size leading to estimates with wide 95% CI’s, limiting multivariable analyses. Second, our analysis does not account for waning antibody levels, which decay over time meaning that our estimation of prior infection may not include points in time early in the pandemic [20]. Third, because we did not interview individuals that declined to participate or did not respond to survey invitation, it is possible that non-response bias may be affecting our findings. For example, it is possible that response rates could be different between individuals that had already previously tested positive for COVID-19 and those that had not. To address non-response bias, we proactively followed up with households that did not respond to the study via telephone calls and home visits. Fourth, this study was conducted using a single assay. Studies have shown that variation in the sensitivity and specificity of the serologic test used in a serosurvey can affect seroprevalence estimates [40]. To limit this effect, we used a test that was previously validated in Massachusetts and conducted a sensitivity analysis using other plausible test sensitivity and specificity values.

## Conclusion

In conclusion, in Holyoke, Massachusetts, a post-industrial, majority Hispanic/Latino/Latina city with high levels of socio-economic disadvantage in MA, USA, seroprevalence of SARS-CoV-2 IgG antibodies was 13.1% at the end of the first year of the pandemic. The risk of infection was higher among the Hispanic/Latino/Latina community, Spanish-speaking households, and communities with high social vulnerability. This knowledge contributed to the city’s targeted public health responses to ensure that high-risk groups would be equitably served. In Holyoke, MA—and in other areas of the USA—disparities in SARS-CoV-2 risk must be addressed through proactive public health interventions that respond to disparities in socially vulnerable communities. These efforts can be supported by rapid, methodologically robust seroprevalence studies undertaken by local boards of health.

## Supplementary Information

Below is the link to the electronic supplementary material.

Supplementary file1 (PDF 668 KB)

## Data Availability

Raw data are available upon reasonable request. Deidentified data can be made available for legitimate research purposes if requested from the senior author (LCI).
