# Supplementary material for: Disparities in SARS-CoV-2 Infection by Race, Ethnicity, Language, and Social Vulnerability: Evidence from a Citywide Seroprevalence Study in Massachusetts, USA
Source: J Racial Ethn Health Disparities. 2023 Jan 18;11(1):110–20. doi: 10.1007/s40615-022-01502-4 (PMC9847437; doi:10.1007/s40615-022-01502-4)
Supplement: Supplementary file 1 — Supplementary file1 (PDF 668 KB) [file 40615_2022_1502_MOESM1_ESM.pdf]

Supplement: Holyoke Seroprevalence Study

1 Bayesian model

1.1 Setup

Following the approach in Stringhini et al. (2020), we use a Bayesian logistic regression model with a random intercept for household clustering. We account for the sensitivity and specificity of the antibody test.

$$
\begin{aligned}
x_i &\sim Bernoulli(p_i * \theta^+ + (1 - p_i) * (1 - \theta^-)) \\
logit(p_i) &= \alpha_h + \beta_0 + \mathbf{Z}_i \boldsymbol{\beta} \\
\alpha_h &\sim Normal(0, \sigma^2) \\
x^+ &\sim Binomial(n^+, \theta^+) \\
x^- &\sim Binomial(n^-, 1 - \theta^-)
\end{aligned}
$$

where x_i is the result of the particular antibody test and p_i is the true underlying probability for the ith person. The sensitivity, θ^+ is determined by n^+ RT-PCR positive controls from the lab validation study with x^+ testing positive (true positive). Similarly, the specificity, θ^- is determined by n^- pre-pandemic controls with x^- testing negative (true negative). The random intercept α_h is indexed by household h. Finally, $\mathbf{Z_i}$ is the collection of covariates we will use for weighting, including age, sex, race ethnicity, and social vulnerability index. This model was implemented using the `rstan` package in R with 5,000 iterations and four chains with 250 warm-up and convergence was assessed using the R-hat statistic. The key difference with the Stringhini et al. (2020) approach is the final weighting step – we used raked weights based on marginal population distributions for numerous variables, whereas Stringhini et al. (2020) used population distributions by age and sex.

1.2 Prior distributions

The prior distributions for θ^+ and θ^- were flat from 0 to 1, equivalent to Uniform(0,1). We used lighter tailed priors for the β coefficients and σ because the No-U-Turn Sampler implemented in `rstan` may have convergence issues with heavy-tailed priors (Ghosh et al., 2018). Notably, we did experience convergence issues when trying Cauchy priors for the β parameters and a flat prior for σ. For the β_0 intercept, we utilized a Normal(0,10) prior. For other β parameters, a Normal (0,2.5) or Normal(0,5) was employed – the latter was used for covariates that were expected to have profound differences in seroprevalence between levels (e.g. occurrence of COVID-19 symptoms, known COVID-19 exposure, suspected or confirmed COVID-19 disease) to ensure we did not push the odds ratios between groups towards null. For σ, we used a truncated Normal prior with mean 1 and standard deviation 1; notably, we tried a half-Normal prior (mean 0) with a standard deviation of 1, with no impact on results.

1.3 Weighting

We first construct population-representative weights based on V categorical variables. In our case, we have $V = 4$ variables: age, race ethnicity, sex, and social vulnerability index (SVI). Subgroups will be denoted based on their variable and level indexed by v and l, respectively. For example, let $V = 1$ correspond to the age variable and $L = 1$ denote persons aged 0-19 years.

Consider partitioning individuals based on each variable into distinct subgroups indexed by j. Let $\mathcal{J}$ be a collection of all distinct subgroups and $\mathcal{I}_j$ denote the collection of indices for all respondents within the distinct subgroup j (e.g. Hispanic females aged 0-19 years living in a high SVI area).

Let $\mathcal{J}_{vl}$ indicate the collection of all distinct subgroups j containing individuals with one common characteristic indicated by variable v and level l. Let $\mathcal{I}_{vl}$ be the collection of indices for all respondents who have the chosen characteristic indicated by variable v and the level l (e.g. $\mathcal{I}_{11}$ would contain the indices of all persons aged 0-19 years in our sample).

The weights were calculated in the `survey` package in R using raking. Raking is an iterative procedure that assigns weights to each individual, w_i, based on the marginal proportions for each variable and level such that:

$$\sum_{i}^{n} w_i = N$$

$$\sum_{i \in I_{vl}} w_i = N_{vl} \text{ for each variable } v \text{ and level } l$$

where n is the sample size, N is the size of the target population, and N_{vl} is the population size of the subgroup

v, l in the target population. For example, N_{11} would be the number of persons aged 0-19 years in Holyoke. Note that raking does not necessarily ensure that $\sum_{i \in I_j} w_i = N_j$; this would only be possible if individual-level data on the City of Holyoke was publicly available. Hence, we rely on the marginal distributions from the American Community Survey in 2019 for each variable (**Table 1**).

Table 1. Holyoke Demographics from American Community Survey 2019

Variable	Proportion
**Age (years)**	
0-19	0.258
20-44	0.357
45-59	0.189
≥ 60	0.196
**Race ethnicity**	
non-Hispanic white	0.413
Hispanic	0.539
non-Hispanic other	0.048
**Sex**	
Female	0.512
Male and other	0.488
**Social vulnerability index**	
Low	0.222
High	0.778

1.4 Overall prevalence

For each iteration r, we take each β_0^r, $\boldsymbol{\beta}^r$, and σ^r and complete the following steps:

Step 1: Compute $p_j^r = \int_\alpha expit(\alpha_h^r + \beta_0^r + \mathbf{Z}_j \boldsymbol{\beta}^r) f_\alpha(\alpha_h^r)$ for each subgroup $j \in \mathcal{J}$

Step 2: Calculate $p^r = \frac{\sum_{j \in \mathcal{J}} (\sum_{i \in \mathcal{I}_j} w_i) p_j^r}{N}$

Let $\mathbf{Z}_j$ be a binary vector aligning with the relevant subgroup j. For Step 1, the integral is computed with a change of variables (see Stringhini paper) and using the `integrate()` function in R. Step 2 approximates the approach used in Stringhini et al. (2020) paper and can be used when cross-tabulations for the target populations are not available. The posterior median (median across all iterations r) is the overall prevalence estimate. The 2.5th and 97.5th percentiles is the 95% credible interval for the overall prevalence.

1.5 Subgroup prevalence

For any subgroup corresponding to variable v and level l,

Step 1: Same as the above

Step 2: Calculate $p_j^r = \frac{\sum_{j \in \mathcal{J}_{vl}} (\sum_{i \in \mathcal{I}_j} w_i) p_j^r}{N_{vl}}$

For a subgroup not included as a weighting variable, let X denote a binary variable. For ease of exposition, let's say interest lies in estimating the prevalence among the subgroup where $X = 1$.

Step 0: Re-run the Bayesian procedure using the following model,

$$logit(p_i) = \alpha_h + \beta_0 + \mathbf{Z}_i \boldsymbol{\beta} + \gamma X$$

Now, for each iteration r, we take each β_0^r, $\boldsymbol{\beta}^r$, γ^r, and σ^r and complete the following steps:

Step 1: Compute probabilities for each distinct weighting subgroup j among persons with $X = 1$,

$$p_{j,X=1}^r = \int_\alpha expit(\alpha_h^r + \beta_0^r + \mathbf{Z}_j \boldsymbol{\beta}^r + \gamma^r) f_\alpha(\alpha_h^r)$$

Step 2: Calculate prevalence within subgroup $X = 1$,

$$p_{X=1}^r = \frac{\sum_{j \in \mathcal{J}} (\sum_{i \in \mathcal{I}_j \cap \mathcal{I}_{X=1}} w_i) p_{j,X=1}^r}{\sum_{j \in \mathcal{J}} \sum_{i \in \mathcal{I}_j \cap \mathcal{I}_{X=1}} w_i}$$

where $\mathcal{I}_{X=1}$ are the indices for persons with $X = 1$.

2 Test sensitivity and specificity considerations

Estimates of test specificity were calculated using 1548 pre-pandemic samples from the Boston area. Figure 1 shows the distribution of IgG antibody levels of the pre-pandemic controls, as well as the corresponding distribution among the serological samples from Holyoke, MA. Estimates of test sensitivity were calculated among individuals not hospitalized with COVID-19 disease (n=94). Disease positive individuals had confirmed RT-PCR tests and up to three blood samples sent for SARS-CoV-2 testing at varying time points after symptom onset. We utilized the second time point for all individuals, which was collected at a median of 28 days after symptom onset and ranged between 6 and 90 days.

The table below gives the sensitivity and specificity estimates for each antibody type and combination. Our main measure used the sensitivity and specificity associated with the presence of IgG antibodies.

Table 2. Sensitivity and specificity estimates used in analyses

Characteristic	n^-	$x^+_{n^-}$	Specificity	n^+	$x^+_{n^+}$	Sensitivity
**IgG**	1548	7	99.5 (99.0-99.8)	94	67	70.6 (61.2-79.3)
**IgM**	1548	9	99.4 (98.9-99.7)	94	38	40.0 (30.0-50.0)
**IgG or IgM**	1548	16	98.9 (98.3-99.3)	94	71	75.0 (65.8-83.2)
**IgG and IgM**	1548	0	99.9 (99.8-100)	94	34	35.7 (26.4-45.7)

We also performed a sensitivity analysis for the overall prevalence using sensitivity estimates corresponding to five other testing time points: first testing date, first testing date if it occurred at or after 14 days, second testing date (above), second testing date if it occurred at or after 14 days, and the third testing date. Interestingly, sensitivity increases the later the test is performed. The table gives the sensitivity estimates for each of these testing time points:

Table 3. Alternate sensitivity estimates for IgG antibodies

Time point	Median (days)	n^+	$x^+_{n^+}$	Sensitivity
**1**	20	96	58	60.2 (50.7-69.6)
**1 ($\geq$ 14 days)**	26	71	53	73.7 (63.4-83.3)
**2**	28	94	67	70.6 (61.2-79.3)
**2 ($\geq$ 14 days)**	28.5	92	67	72.1 (62.8-80.8)
**3**	47	84	67	78.8 (69.7-86.6)

We implemented the Bayesian procedure to estimate overall prevalence for the varying sensitivity estimates presented in Table 3. The results are plotted in Figure 2.

Figure 1: Distribution of IgG titer values among pre-pandemic samples (top) and serological samples from Holyoke, MA(bottom).

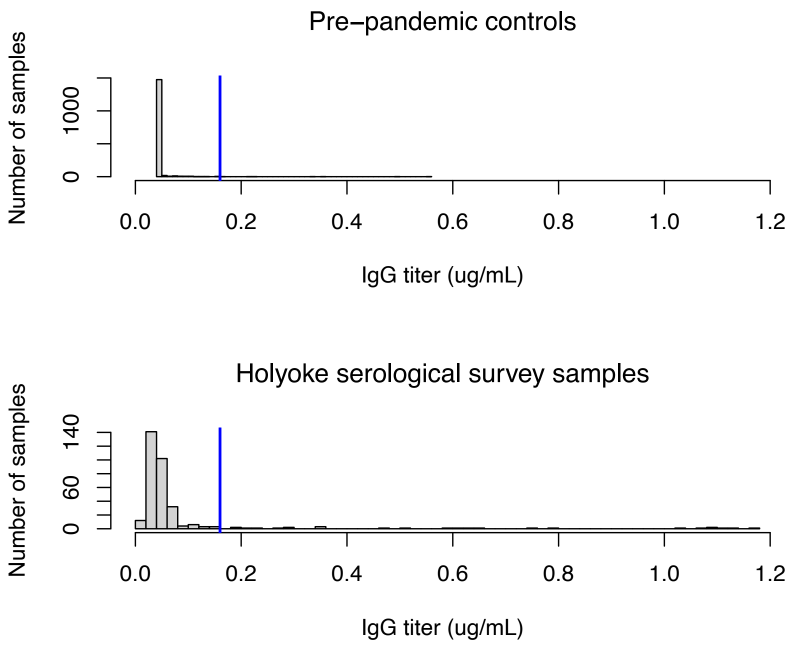

Figure 2: Estimated seroprevalence as a function of test sensitivity.

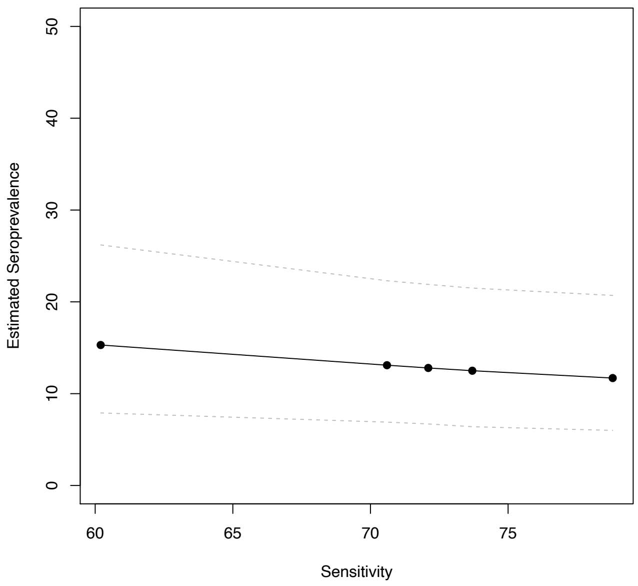

3 Sample Size Statement

Random sampling was employed as we had no baseline information on response rates by census tract. Our original sample size calculation assumed perfect correlation in SARS-CoV-2 infection within a household. Notably, this was a conservative estimate as there were no great estimates of the intracluster correlation coefficient (ICC) in May 2020. We assumed the population prevalence was 15% and would thus need to enroll at least 400 households to have a 95% confidence interval width of 7%. The average household size in Holyoke is 2.3, so we estimated this would translate to 920 individuals. We inflated the number of households by response rate (25-30%) and vacant lot rate of 5%. Our assumed household response rate was higher than what we achieved.

Supplementary Table 1. Sociodemographic, symptom, testing and exposure history stratified by race/ethnicity.

	Hispanic, Latino/Latina	White, non-Hispanic	Grouped race/ethnicity, non-Hispanic
**Characteristic (N if not 328[1])**	66	239	23
**Age group (years) (%)**			
0-19	12 (18.2)	14 (5.9)	1 (4.3)
20-44	14 (21.2)	54 (22.6)	8 (34.8)
45-59	24 (36.4)	64 (26.8)	6 (26.1)
60-84	15 (22.7)	100 (41.8)	8 (34.8)
85+	1 (1.5)	7 (2.9)	0 (0.0)
**Age (years) (median [IQR])**	53.00 [31.50, 59.00]	57.00 [39.00, 68.00]	50.00 [35.50, 62.00]
**Gender (%)**			
Female	27 (40.9)	102 (42.7)	10 (43.5)
Male	39 (59.1)	130 (54.4)	11 (47.8)
Trans woman	0 (0.0)	1 (0.4)	0 (0.0)
Non-binary	0 (0.0)	5 (2.1)	2 (8.7)
Prefer not to answer	0 (0.0)	1 (0.4)	0 (0.0)
**Primary language spoken in the household (%) (N = 327)**			
English	20 (30.3)	234 (98.3)	21 (91.3)
Spanish	35 (53.0)	1 (0.4)	0 (0.0)
Multi-lingual	10 (15.2)	2 (0.8)	1 (4.3)
Other	1 (1.5)	1 (0.4)	1 (4.3)
**Highest education level (%) (N=298)***			
Some high school or less	16 (32.7)	5 (2.2)	1 (4.8)
High school/GED or some college	18 (36.7)	74 (32.5)	3 (14.3)
Associate or bachelor's degree	13 (26.5)	78 (34.2)	10 (47.6)
Master's doctorate or professional degree	2 (4.1)	71 (31.1)	7 (33.3)
**Employment status on February 1st, 2020 (%) (N=304)***			
Not Working	17 (31.5)	73 (32.0)	4 (18.2)
Working	22 (40.7)	142 (62.3)	17 (77.3)
Other	15 (27.8)	13 (5.7)	1 (4.5)
**Salary impacted by COVID-19 (%) (N=304)***			
No	31 (57.4)	165 (72.4)	14 (63.6)
Yes	23 (42.6)	63 (27.6)	8 (36.4)
**Worked outside home during "stay at home" order (%) (N=304)***			

No	40 (74.1)	154 (67.5)	17 (77.3)
Yes	14 (25.9)	74 (32.5)	5 (22.7)
**Worked as a health worker in a healthcare setting (%) (N=93)****			
No	12 (85.7)	59 (79.7)	4 (80.0)
Yes	2 (14.3)	15 (20.3)	1 (20.0)
**Workplace offered paid sick leave, work-from-home, overtime, or hazard pay during Mar – June (%) (N=93)*⁺**			
1 or 2	4 (28.6)	49 (66.2)	3 (60.0)
3 or 4	1 (7.1)	5 (6.8)	0 (0.0)
None	9 (64.3)	20 (27.0)	2 (40.0)
**Could you afford to stay home sick from work? (%) (N=92)****			
No	6 (42.9)	7 (9.6)	2 (40.0)
Yes	8 (57.1)	66 (90.4)	3 (60.0)
**Have you had any symptoms since Feb, 2020? (%) *****			
No	26 (39.4)	116 (48.5)	13 (56.5)
Yes	40 (60.6)	123 (51.5)	10 (43.5)
**COVID-19 testing history (%) (N=326)**			
I have always tested negative	25 (38.5)	107 (44.8)	9 (40.9)
I have never been tested for COVID-19	35 (53.8)	120 (50.2)	12 (54.5)
I tested positive at least once	5 (7.7)	12 (5.0)	1 (4.5)
**Exposure to individual with COVID-19? (%) (N=327)**			
I have no known exposure to COVID-19	50 (75.8)	186 (78.2)	20 (87.0)
I was exposed to a household member with COVID-19	6 (9.1)	10 (4.2)	0 (0.0)
I was exposed to a non-household member with COVID-19	10 (15.2)	42 (17.6)	3 (13.0)
**Exposed to individual with respiratory illness? (%) (N=326)**			
Household member	5 (7.6)	6 (2.5)	0 (0.0)
No known exposure	58 (87.9)	204 (86.1)	20 (87.0)
Non-household member	3 (4.5)	27 (11.4)	3 (13.0)
**Have you ever been contacted by a contact tracer? (%) (N=327)**			
No	66 (100.0)	228 (95.8)	23 (100.0)
Yes	0 (0.0)	10 (4.2)	0 (0.0)
**Did you use the bus since Feb 2020? (%) (N=323)**			
No	62 (93.9)	230 (97.9)	22 (100.0)
Yes	4 (6.1)	5 (2.1)	0 (0.0)

**Travel since February 1st, 2020 (%) (N=326)**			
Different city	8 (12.1)	99 (41.6)	10 (45.5)
Different country	4 (6.1)	3 (1.3)	0 (0.0)
Different state	14 (21.2)	71 (29.8)	3 (13.6)
No	40 (60.6)	65 (27.3)	9 (40.9)
**Home type (%) (N=326)**			
Single Family	12 (18.2)	165 (69.6)	12 (52.2)
Multi-family	18 (27.3)	42 (17.7)	7 (30.4)
Apartment or condominium	35 (53.0)	28 (11.8)	4 (17.4)
Other	1 (1.5)	2 (0.8)	0 (0.0)
**Rent or own the home (%) (N=323)**			
Someone in the household owns house	33 (50.0)	189 (80.4)	13 (59.1)
We have a landlord	33 (50.0)	46 (19.6)	9 (40.9)
**Number of household members (mean (SD))**	3.30 (2.31)	2.37 (1.07)	2.22 (0.80)
**Annual household income (%) (N=325)**			
Less than $15,000	15 (22.7)	5 (2.1)	1 (4.3)
$15,000 - $50,000	19 (28.8)	44 (18.6)	6 (26.1)
$50,000 - $80,000	8 (12.1)	50 (21.2)	6 (26.1)
$80,000 - $160,000	4 (6.1)	84 (35.6)	6 (26.1)
>$160,000	0 (0.0)	23 (9.7)	1 (4.3)
Prefer not to answer	20 (30.3)	30 (12.7)	3 (13.0)
**Do you have a computer with internet at home? (%) (N=322)**			
No	24 (39.3)	10 (4.2)	0 (0.0)
Yes	37 (60.7)	228 (95.8)	23 (100.0)
**In the past 6 months, has your HH received any public assistance? (%) (N=3223)******			
No assistance	1 (1.5)	47 (20.0)	7 (30.4)
1 type of assistance	12 (18.5)	89 (37.9)	6 (26.1)
2 - 3 types of assistance	39 (60.0)	91 (38.7)	10 (43.5)
4 - 5 types of assistance	13 (20.0)	8 (3.4)	0 (0.0)

[1]Respondents excluded if missing response for a certain variable

*Among adult respondents only.

**Among individuals that responded "Yes" to working outside the home during the "stay at home" order.

***Symptoms include cough, sore throat, chills, shortness of breath, fever, body aches, nausea, loss of smell, loss of appetite, fatigue, headache.

****Assistances include: WIC, TANF, SSI, Public medical insurance, unemployment insurance, COVID-19 related stimulus payment, Food pantry/Food basket support.

5 **Figure 3. Timing of survey completion and dried blood spot sample collection.**

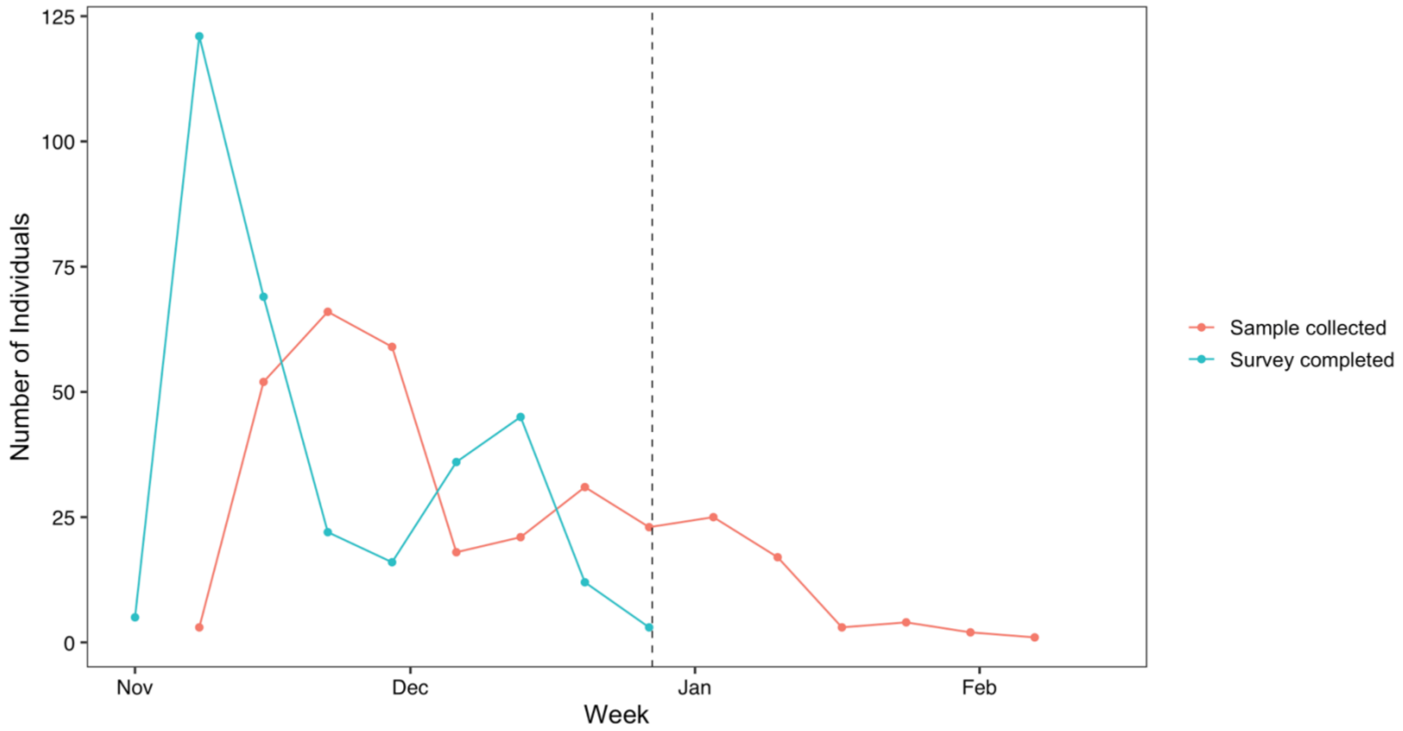

By 12/28/2020, the date of the survey deadline, 82% of samples had already been collected
By 1/15/2021, 96% of samples had been collected. Only 1% (n=3) samples were collected after 1/31/2021.
